# Prevalence of Bacterial Coinfections with *Vibrio harveyi* in the Industrialized Flow-through Aquaculture Systems in Hainan Province: A Neglected High-Risk Lethal Causative Agent to Hybrid Grouper

**DOI:** 10.3390/ijms231911628

**Published:** 2022-10-01

**Authors:** He Xu, Yan-Hua Zeng, Wen-Liang Yin, Hong-Bin Lu, Xiao-Xiao Gong, Na Zhang, Xiang Zhang, Hao Long, Wei Ren, Xiao-Ni Cai, Ai-You Huang, Zhen-Yu Xie

**Affiliations:** 1 State Key Laboratory of Marine Resource Utilization in the South China Sea, Hainan University, Haikou 570228, China; 2 Laboratory of Development and Utilization of Marine Microbial Resource, Hainan University, Haikou 570228, China; 3 College of Marine Sciences, Hainan University, Haikou 570228, China; 4 Key Laboratory of Tropical Hydrobiology and Biotechnology of Hainan Province, Haikou 570228, China

**Keywords:** bacterial coinfections, *Vibrio harveyi*, hybrid grouper, artificial infection, industrialized aquaculture

## Abstract

*Vibrio harveyi* is one of the most serious bacterial pathogens to aquatic animals worldwide. Evidence is mounting that coinfections caused by multiple pathogens are common in nature and can alter the severity of diseases in marine animals. However, bacterial coinfections involving *V. harveyi* have received little attention in mariculture. In this study, the results of pathogen isolation indicated that bacterial coinfection was a common and overlooked risk for hybrid groupers (♀ *Epinephelus polyphekadion* × ♂ *E. fuscoguttatus*) reared in an industrialized flow-through pattern in Hainan Province. The artificial infection in hybrid groupers revealed that coinfections with *V. harveyi* strain GDH11385 (a serious lethal causative agent to groupers) and other isolated pathogens resulted in higher mortality (46.67%) than infection with strain GDH11385 alone (33.33%), whereas no mortality was observed in single infection with other pathogens. Furthermore, the intestine, liver and spleen of hybrid groupers are target organs for bacterial coinfections involving *V. harveyi*. Based on the infection patterns found in this study, we propose that *V. harveyi* may have a specific spatiotemporal expression pattern of virulence genes when infecting the host. Taken together, bacterial coinfection with *V. harveyi* is a neglected high-risk lethal causative agent to hybrid groupers in the industrialized flow-through aquaculture systems in Hainan Province.

## 1. Introduction

Bacterial coinfections are very common in nature and occur when two or more pathogens invade one host by simultaneous or secondary infections [1,2]. However, due to the complexity of interactions between different pathogens and between pathogens and their hosts [3,4,5], our understanding on the prevalence of bacterial coinfections in aquatic animals such as fish and the mechanisms of infection are still limited [6]. Coinfections associated with opportunistic pathogens tend to be more harmful to aquatic animals than nonopportunistic pathogens, since opportunistic pathogens are often able to persist and spread in the environment [7]. Opportunistic pathogens with broad host ranges are more likely to find potential hosts than host-specific pathogens [8]. Moreover, highly virulent strains were found to have enhanced interference competition, and these strains are likely to have a competitive advantage in aquaculture settings [9]. Therefore, opportunistic pathogens present a high risk of causing coinfections, which largely limits the development of some aquaculture industries [9].

The hybrid grouper (♀ *Epinephelus polyphekadion* × ♂ *E. fuscoguttatus*) is a new artificial species that has been widely farmed in South China [10]. Taking Hainan Province as an example, the aquaculture area of groupers is as high as 12,000 mu, with an annual output of 100,000 tons. In recent years, the culture of hybrid groupers in Hainan Province is mainly based on an industrialized flow-through reared pattern [11]. Unfortunately, the survival rate of industrialized reared hybrid groupers in Hainan Province fluctuates between 20~90%; in 2021 in particular, the survival rate was less than 40%. One of the major reasons for the decline in the survival rate of hybrid groupers is the outbreak of bacterial diseases. It is particularly worth noting that *Vibrio* is one of the most harmful bacterial pathogens in the industrialized reared hybrid grouper in Hainan Province [6].

One of the most notorious opportunistic pathogens, *Vibrio harveyi*, has been reported frequently since the 1990s to infect a wide range of marine fish and invertebrates [12,13,14,15,16,17,18,19]. Various tissues or organs, such as the brain [20], gills [21], eyeball [22], liver [19], spleen [23], head kidney [24], intestine [25,26] and heart [27] have been reported to be targets of *V. harveyi* infection. As one of the most common pathogens worldwide, the crucial virulence genes of *V. harveyi* are still not systematically revealed [28,29] since each step of its infection is usually tightly controlled by specific virulence factors [30]. Therefore, analyzing the infection characteristics under different infection patterns, such as coinfections and monoinfection, is of great significance to uncover the key virulence genes. However, little information is currently available on the infection patterns of *V. harveyi*.

Our previous study demonstrated that the isolate GDH11385 was the dominant genotypic strain of the lethal pathogen *V. harveyi*, which infects the hybrid grouper and other aquaculture animals in South China [11]. A recent report has also shown that coinfection with *V. alginolyticus* and *V. harveyi* caused a more devastating impact on farmed fish than the monoinfection with *V. alginolyticus* or *V. harveyi* [31]. Therefore, we speculate that the coinfections with *V. harveyi* and other bacterial pathogens may have a potentially high lethal risk for hybrid groupers in the industrialized flow-through reared pattern in Hainan Province. To test this hypothesis, this study investigated the prevalence of bacterial coinfections in hybrid groupers in the current industrialized flow-through reared pattern in Hainan Province. Artificial infection experiments were conducted in healthy hybrid groupers with *V. harveyi* strain GDH11385 and the isolated dominant pathogens under both coinfections and monoinfection modes, and then the mortality rate was compared. In addition, we explored the infection pattern of *V. harveyi* in hybrid groupers by counting the number of pathogens in the infected organs.

## 2. Results

### 2.1. Predominant of Vibrio spp. in the Liver of Diseased Hybrid Groupers

**Figure 1:**
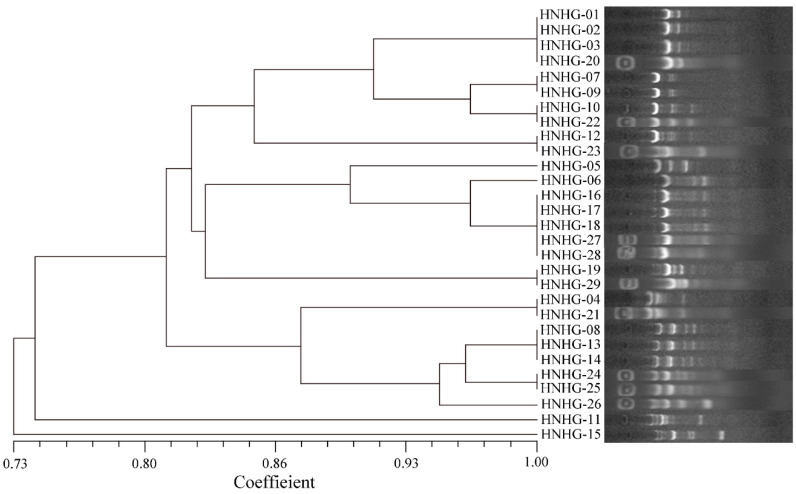
Dendrogram of isolated pathogens from liver samples of the diseased hybrid groupers based on ERIC-PCR analysis.

No bacterial strains were isolated from unused aquaculture water, indicating that there were no pathogenic bacteria in the unused aquaculture water. For the diseased hybrid groupers, a total of 29 strains were isolated (Table 1). As shown in the result of ERIC-PCR (Figure 1), there was a certain consistency among pathogens of different diseased hybrid groupers. The similarity between HNGN-01, 02, 03 and 20 was 100%. There was also 100% similarity between HNGN-16, 17, 18, 27 and 28; between HNGN-07 and 09; between HNGN-10 and 22; between HNGN-12 and 23; between HNGN-19 and 29; and between HNGN-04 and 21. Combining the results of 16S rRNA gene sequencing and ERIC-PCR typing, 12 distinct strains were identified (Figure 1; Table 1). As revealed in Table 1, 20 strains were affiliated to the genus *Vibrio*, including strain HNGN-01, 02, 03, 04, 05, 07, 08, 09, 10, 11, 12, 13, 14, 20, 21, 22, 23, 24, 25 and 26, which accounted for 68.97% of the total strains. According to the statistics of the quantity proportion of all isolated strains, the quantity proportion of *V. proteolyticus* NBRC 13287, *V. owensii* LMG 25443, *V. ponticus* CECT 5869 and *Shewanella corallii* fav-2-10-05 in all isolated strains was more than 10%. Based on the results of bacterial isolation, ERIC-PCR typing and 16S rRNA gene sequencing, the distribution of pathogens in diseased hybrid groupers had a certain diversity, and multiple bacterial coinfections of hybrid groups existed in the Hainan industrialized flow-through aquaculture systems.

### 2.2. Higher Mortality Rate in Coinfections with V. harveyi GDH11385 Than Monoinfection

To confirm whether coinfections with *V. harveyi* GDH11385 and three isolated pathogens) had higher mortality rates than monoinfections of strain GDH11385, we injected mixed fluids of isolated pathogens and different concentrations of GDH11385 into the healthy hybrid groupers. The mortality rates of injected hybrid groupers were measured at different challenge concentrations (Table 2). No death occurred in either group A or group CK. When strain GDH11385 coinfected the healthy hybrid groupers with other pathogens, the death of hybrid groupers began to occur. The 7-day average mortality gradually increased as the concentration of *V. harveyi* GDH11385 rose. In group B (GDH11385: 1 × 10^3^ CFU/g), the 7-day average mortality of hybrid groupers was 3.33%; in group C (GDH11385: 1 × 10^4^ CFU/g), the 7-day average mortality of hybrid groupers was 13.33%; in group D (GDH11385: 1 × 10^5^ CFU/g), the 7-day average mortality of hybrid groupers was 46.67%. In group E, when strain GDH11385 infected the healthy hybrid groupers alone at the same concentration as in group D, the 7-day average mortality of hybrid groupers dropped to 33.33%.

### 2.3. The Duration of the Coinfection State Depends on the Infection Concentration of Strain GDH11385

**Figure 2:**
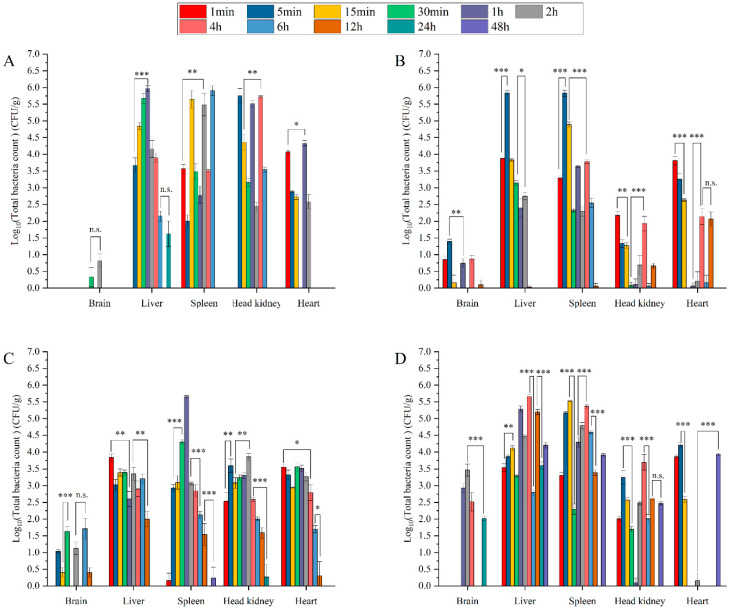
Spatiotemporal distribution of coinfected pathogens in brain, liver, spleen, head kidney and heart over 48 h postinjection. Data are shown as mean ± SD of three independent biological replicates. n.s., not significant; * *p* < 0.05, ** *p* < 0.01, *** *p* < 0.001. (**A**–**D**), Pathogen distribution of group A (**A**), group B (**B**), group C (**C**) and group D (**D**).

To explore the distribution of multiple pathogens under different challenge concentrations of strain GDH11385, we counted the total bacterial numbers in the five organs of the injected hybrid grouper to observe the changes in the infection process. Compared with group A, the change trend of the total number of colonies in the five organs of groups B, C and D was basically the same. The total number of colonies in groups A and B decreased rapidly and almost disappeared at 12 h postinfection (hpi) (Figure 2A,B). In group C, *Vibrio* almost completely disappeared at 24 hpi (Figure 2B). Although the total number of bacterial colonies in group D changed significantly, the total number of pathogens in the three organs of the intestine, liver and spleen was still relatively large (Figure 2A–D).

### 2.4. Intestine, Liver and Spleen Were Target Organs of GDH11385 in Coinfections and Monoinfection

**Figure 3:**
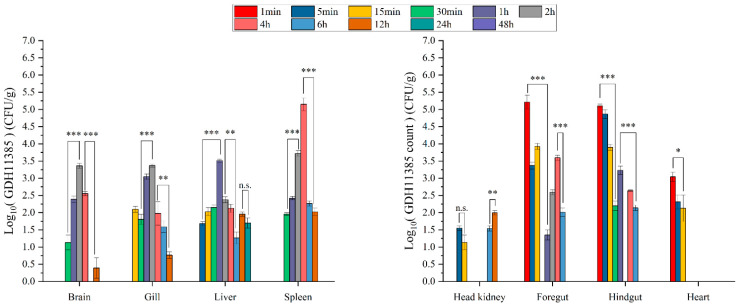
The distribution of GDH11385 in brain, liver, spleen, head kidney, gills, foregut, hindgut and heart of coinfected hybrid groupers (group D) over 48 h postinjection. Data are shown as mean ± SD of three independent biological replicates. n.s., not significant; * *p* < 0.05, ** *p* < 0.01, *** *p* < 0.001.

According to the results of the change trend of the total number of colonies, we selected groups D and E for further analysis to observe the effects of multiple pathogens on the infection of strain GDH11385. In the progression of monoinfection, strain GDH11385 was first detected in the foregut, hindgut and heart at 1 min postinjection (mpi), in the liver and head kidney at 5 mpi, and in the brain, gills and spleen at 15 mpi (Figure 3). In the early stage of infection, the load of strain GDH11385 in the intestine was relatively stable and then declined at 30 mpi, while the load of GDH11385 in the brain, liver, spleen, head kidney, gills and heart increased at 5–30 mpi.

**Figure 4:**
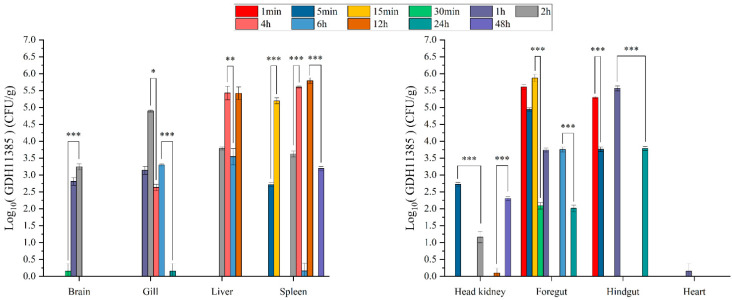
The distribution of GDH11385 in brain, liver, spleen, head kidney, gills, foregut, hindgut and heart of monoinfected hybrid groupers (group E) over 48 h postinjection. Data are shown as mean ± SD of three independent biological replicates. n.s., not significant; * *p* < 0.05, ** *p* < 0.01, *** *p* < 0.001.

**Figure 5:**
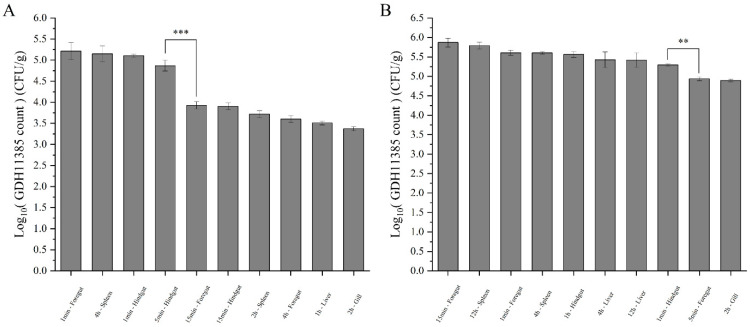
Comparison of maximum GDH11385 burden in brain, liver, spleen, head kidney, gills, foregut, hindgut and heart over 48 h postinjection. (**A**) Ten data with the maximum GDH11385 burden in group D; (**B**) ten data with the maximum GDH11385 burden in group E. Data are shown as mean ± SD of three independent biological replicates. ** *p* < 0.01, *** *p* < 0.001.

In the progression of coinfections with strain GDH11385 and three isolated pathogens, strain GDH11385 was first detected in the foregut and hindgut at 1 mpi, in the spleen and head kidney at 5 mpi, in the brain and gills at 15 mpi and in the liver at 2 hpi (Figure 4). The trend of GDH11385 load in the intestine at the initial stage of infection was consistent with the progression trend in monoinfection, while the load of GDH11385 increased in the brain, liver, spleen, head kidney, gills and heart at 1–2 hpi. Based on the above results (Figure 5A,B), the load of strain GDH11385 was more enriched in some organs such as the intestine (foregut and hindgut), liver and spleen during the progress of coinfection and monoinfection.

## 3. Discussion

Coinfections are defined as the infection of a host with two or more different pathogens [1,32]. However, this topic has received little attention in industrialized reared hybrid groupers, even though such infections are very common in nature. In this study, we conducted an epidemiological survey and found that coinfections with multiple pathogens are prevalent in the industrialized reared hybrid groupers in Hainan Province. Among the isolated strains, strains of genus *Vibrio* were the main pathogens coinfecting hybrid groupers. Typically, the virulence of coinfecting pathogens is determined by interactions, reciprocity or competition between coinfecting bacterial strains [33,34,35]. However, we noticed that the dominant pathogens isolated in this study did not cause the death of healthy hybrid groupers through artificial infection, which means that the isolated multiple pathogens could successfully invade the host, but were not lethal to the host. The results of this study showed that the mortality of injected groupers was only observed in the groups of monoinfection or coinfections with *V. harveyi* strain GDH11385, which is the major genotypic strain of lethal pathogens in aquaculture animals in South China, including Hainan Province [11]. In addition, the mortality of coinfection with strain GDH11385 and isolated pathogens was more than 46% higher than that of GDH11385 infection alone. These results suggest that *V. harveyi* strains such as GDH11385 are a potential determinant of increased coinfection virulence and subsequent mortality of groupers in the current industrialized aquaculture systems.

To avoid the monoinfection or coinfection of *V. harveyi*, we should enforce the supervision of *V. harveyi* in the industrialized reared system. Although we have established a PCR-based technique for the detection of *V. harveyi* [11], the sensitivity and applicability of this technique are still insufficient. Therefore, efforts should be made to improve the accuracy and sensitivity of the detection technique for *V. harveyi* in future research.

Combining the results of total bacterial colony counts and the mortality of groupers in the coinfection experiment, we found that the higher the initial amount of strain GDH11385 in the coinfections, the longer the duration of the coinfection process; meanwhile, the mortality of groupers also gradually increased. This suggested that the duration of *V. harveyi* infection in the hybrid groupers during the coinfection progress was one of the most important factors in the increased lethality of *V. harveyi*. The ultimate strength of virulence is an important basis for the bacterial infection process, whether it is a monoinfection or a coinfection process [36]. Importantly, the virulence is related to the invasiveness and toxin of bacteria [37], and the invasiveness is related to the speed and duration of bacterial colonization and infection [38]. In most cases, the fish hosts infected with *Vibrio* pathogens usually do not die rapidly due to pathogen–host interactions. Iron acquisition, as a fierce battle occurring between pathogenic *Vibrio* and the fish host, is a pivotal step for virulence. For example, siderophores have been shown to be relevant virulence factors critical for colonization of the fish hosts [39]. To overcome the iron limitation by the host, *Vibrio* pathogens often take up iron from the hosts via siderophores. *Vibrio* requires iron to increase its pathogenicity, which is why it takes a longer time for morbidity and even mortality, meaning that death occurs when there are as many bacteria as possible in the tissues. Therefore, based on our study, we speculate that when the major lethal pathogen *Vibrio harveyi* GDH11385 is involved in the coinfection process, pathways related to pathogen invasiveness make a greater contribution to persistent infection with pathogen, further elevating the host mortality.

Based on the distribution of *V. harveyi* GDH11385 in the organs of the tested animal, we found that the infection pattern of strain GDH11385 to hybrid groupers occurred in a predetermined order. The intestine was infected at 1 mpi, followed by the liver at 5 mpi, the gills at 15 mpi and then the brain and spleen at 0.5 hpi. According to the spatiotemporal distribution of strain GDH11385 in the infected organs of the host, the intestine, liver and spleen were the main infected organs both in the monoinfection and coinfections. More specifically, the total *Vibrio harveyi* GDH11385 counts (TVC) in both spleen and liver showed two peaks within 24 h, with TVC peaks in spleen appearing at 4 hpi and 12–24 hpi, while TVC peaks in liver were observed at 1–4 hpi and 12 hpi. This pattern of liver infection is similar to that observed in some aquaculture animals infected by *V. harveyi* [23,40,41,42]. In summary, there are obvious spatiotemporal changes in the process of *V. harveyi* infecting the host. In the infection pattern of *Pseudomonas plecoglossicida*, all tested organs of the hybrid grouper except blood (1 mpi) were infected at 0.5–1 hpi, and the load peak of TVC was observed at 24 h [43,44]. Defoirdt indicated that each step of infection was controlled by a number of specific virulence factors that enable pathogens to cause infection, propagation and disease [30]. Therefore, we speculate that *V. harveyi* might have a specific spatiotemporal expression pattern of the virulence genes while infecting the host.

In conclusion, bacterial coinfections with *V. harveyi* are common in the current industrialized flow-through aquaculture systems in Hainan Province, which may be a neglected high-risk lethal causative agent to hybrid groupers. Importantly, the infection pattern of organs by *V. harveyi* strain GDH11385 occurred in an established order. Based on the speculation in this paper, we will conduct further studies on the spatiotemporal expression pattern of virulence genes and invasiveness-related pathways in *V. harveyi*, to find the key virulence genes of *V. harveyi* and understand the reasons for the virulence formation of high-virulence strains of *V. harveyi*.

## 4. Materials and Methods

### 4.1. Isolation and Identification of Bacterial Pathogens from Diseased Hybrid Groupers

Bacterial strains were isolated from unused aquaculture seawater and liver samples of diseased hybrid groupers. The liver samples of ten diseased hybrid groupers were ground into homogenate using an OSE-Y50 Tissue Grinder (Tiangen, Beijing, China). The homogenate and unused aquaculture seawater were diluted 100-fold with sterilized seawater, and the dilution was then spread on thiosulfate-citrate-bile salts-sucrose (TCBS) agar medium. After 24 to 48 h of incubation at 28 °C, morphologically different colonies were picked up and streaked onto new TCBS agar plate to obtain pure cultures.

Both 16S rRNA gene and enterobacterial repetitive intergenic consensus (ERIC) sequence of bacterial strains were amplified for molecular identification. Amplification of the 16S rRNA gene was performed with the universal bacterial primers 27F (5′-AGAGTTTGATCCTGGCTCAG-3′) and 1492R (5′-GGTTACCTTGTTACGACTT-3′) [45]. The primers used for ERIC-PCR were ERIC-F (5′-ATGTAAGCTCCTGGGGATTCAC-3′) and ERIC-R (5′-AAGTAAGTGACTGGGGTGAGCG-3′) [46]. The PCR reactions were performed in a T100 thermal cycler (Hercules, CA, USA) using a Green Taq Mix (Vazyme, Nanjing, China). The PCR conditions for 16S rRNA gene were as follows: 95 °C for 5 min; 30 cycles of 94 °C for 30 s, 56 °C for 30 s and 72 °C for 2 min; and a final extension at 72 °C for 10 min. The cycling conditions for ERIC-PCR were as follows: 95 °C for 7 min; 35 cycles of 90 °C for 30 s, 52 °C for 1 min and 65 °C for 8 min; and a final extension at 68 °C for 16 min. All the ERIC-PCR products were resolved by 1.5% agarose gel electrophoresis. Amplified DNA fragments obtained from the agarose gel electrophoreses were recorded. The observed bands in the gels were evaluated based on the presence (encoded 0) or absence (encoded 1) of polymorphic fragments for the ERIC primers. Cluster analysis was performed with NTSYS-pc (Version 2.10), a numerical taxonomy and multivariate analysis software package, based on Dice’s similarity coefficient (SD) with a 1% position tolerance and the unweighted pair group method using arithmetic averages (UPGMA). The purified 16S rRNA gene fragments were sent to BGI-Tech (Shenzhen, China) for sequencing. The obtained 16S rRNA gene sequence was compared with closely related sequences of reference species from the EzTaxon-e server (https://www.ezbiocloud.net/identify) (accessed on 5 May 2021) [47].

### 4.2. Preparation of Bacterial Cultures

*V. harveyi* strain GDH11385 was isolated from diseased groupers in an aquaculture system in our previous work [11]. Strain GDH11385 was routinely grown overnight at 30 °C in Trypticase Soy Broth (TSB) liquid medium. For the isolated pathogens, strains with colonies greater than 10% of the total colonies of all strains were selected for subsequent artificial infection experiment. These selected isolates were each cultured overnight at 30 °C in 2216E liquid medium, after which they were washed three times and diluted to the same concentration with sterile 1 × phosphate-buffered saline (PBS). According to the statistics of the quantitative ratio of all isolated pathogens, the pathogens with a quantitative ratio greater than 10% were selected for artificial infection.

### 4.3. Artificial Infection

Healthy hybrid groupers (♀ *Epinephelus polyphekadion* × ♂ *E. fuscoguttatus*) were used for artificial infection. All healthy hybrid groupers were obtained from a commercial farm in Wenchang City, Hainan Province, China (body weight = 12 ± 1.0 g). The hybrid groupers were cultured in ten 100 L tanks (length × width × height = 63.5 cm × 42 cm × 40 cm) with an internal water depth of about 30 cm. The seawater used for aquaculture was filtered and sanitized, the water temperature was always controlled at 28–30 °C and the water salinity was maintained at 30‰. A total of 400 healthy hybrid groupers were divided into 5 experimental groups (groups A, B, C, D and E) with 80 hybrid groupers in each group. The control group consisted of 50 healthy hybrid groupers (group CK).

The bacterial culture of strain GDH11385 was diluted to three concentrations: 1 × 10^5^, 1 × 10^6^ and 1 × 10^7^ CFU/mL. Cultures of the selected pathogens were individually diluted to 1 × 10^7^ CFU/mL and were then mixed in equal volumes. For artificial infection, the concentration of bacterial fluid injected into fish in each experimental group is shown in Table 1. Specifically, fish in groups A, B, C and D were intraperitoneally injected with 100 µL of a mixed bacterial suspension of the isolated pathogens. At the same time, fish in groups B, C and D were also injected with 100 µL of bacterial suspension of strain GDH11385 with concentrations of 1 × 10^5^, 1 × 10^6^, and 1 × 10^7^ CFU/mL, respectively. The final concentration of strain GDH11385 in groups B, C and D reached 1 × 10^3^, 1 × 10^4^ and 1 × 10^5^ CFU/g, respectively. Fish in group E were injected with 100 µL of bacterial suspension of strain GDH11385 (final concentration 1 × 10^5^ CFU/g) and 100 µL of 1 × PBS buffer. Fish in group A and group CK were injected with 100 µL and 200 µL of 1 × PBS buffer, respectively. The injected fish in each group were separately put back into clean tanks and cultured at 28–30 °C in seawater.

In each group, 30 injected fish were divided into three parallel groups. These injected fish were cultured individually to observe mortality within 7 days. After injection, we randomly selected three fish from groups A, B, C, D and E, and collected eight organs (brain, liver, spleen, head kidney, gills, foregut, hindgut and heart) at each time point (1 min, 5 min, 15 min, 30 min, 1 h, 2 h, 4 h, 6 h, 12 h, 24 h, 48 h postinjection) for total bacterial colony counting.

### 4.4. Total Bacterial Colony Counting

For total bacterial colony counting, 0.5 mL of 3% saline was added to each organ and then ground into homogenate. After the homogenate was layered, 100 μL of the supernatant was collected and diluted 1 × 10^2^-, 1 × 10^3^- and 1 × 10^4^-fold, sequentially. Then, 50 μL of each dilution was spread on TCBS agar medium (*n* > 3). Bacteria were grown on TCBS agar medium for 48 h.

### 4.5. Colony Identification of Strain GDH11385

To assess the colony number of *V. harveyi* strain GDH11385, we used specific primers for identification. The number of positive PCR results can represent the colony number of strain GDH11385 grown on TCBS agar plates. Amplification of strain GDH11385-specific gene fragment was carried out using primers 3858P1-F (5′-TACATCTACCATTTCCGTAAACATG-3′) and 3858P1-R (5′-ATTGAGGTAAAAGTTTCAGCACATC-3′) [11]. The PCR conditions were as follows: 95 °C for 5 min; 30 cycles of 95 °C for 1 min, 60 °C for 30 s and 72 °C for 1 min; and a final extension at 72 °C for 10 min. The target length of strain GDH11385-specific gene was 656 bp.

## Figures and Tables

**Table 1:** 16S rRNA gene sequencing and alignment results of strains isolated from the livers of diseased hybrid groupers.

Isolates	Top-Hit Taxon and Strain	Similarity (%)
HNHG-01	*Vibrio proteolyticus* NBRC 13287	99.22
HNHG-02	*Vibrio proteolyticus* NBRC 13287	99.26
HNHG-03	*Vibrio proteolyticus* NBRC 13287	99.27
HNHG-04	*Vibrio fortis* LMG 21557	99.86
HNHG-05	*Vibrio harveyi* NBRC 15634	99.79
HNHG-06	*Shewanella corallii* fav-2-10-05	99.07
HNHG-07	*Vibrio owensii* LMG 25443	99.86
HNHG-08	*Vibrio ponticus* CECT 5869	99.72
HNHG-09	*Vibrio owensii* LMG 25443	99.86
HNHG-10	*Vibrio hyugaensis* 090810a	99.78
HNHG-11	*Vibrio parahaemolyticus* NBRC 12711	99.43
HNHG-12	*Vibrio taketomensis* C4III282	99.93
HNHG-13	*Vibrio owensii* LMG 25443	99.86
HNHG-14	*Vibrio ponticus* CECT 5869	99.86
HNHG-15	*Photobacterium damselae* subsp. *piscicida* NCIMB 2058	99.16
HNHG-16	*Shewanella corallii* fav-2-10-05	99.14
HNHG-17	*Shewanella corallii* fav-2-10-05	98.81
HNHG-18	*Shewanella corallii* fav-2-10-05	99.00
HNHG-19	*Providencia rettgeri* DSM 4542	99.64
HNHG-20	*Vibrio proteolyticus* NBRC 13287	99.20
HNHG-21	*Vibrio fortis* LMG 21557	99.78
HNHG-22	*Vibrio hyugaensis* 090810a	99.33
HNHG-23	*Vibrio taketomensis* C4III282	99.86
HNHG-24	*Vibrio rhodolitus* G98	99.86
HNHG-25	*Vibrio ponticus* CECT 5869	99.92
HNHG-26	*Vibrio ponticus* CECT 5869	99.86
HNHG-27	*Shewanella corallii* fav-2-10-05	99.85
HNHG-28	*Shewanella corallii* fav-2-10-05	99.48
HNHG-29	*Providencia rettgeri* DSM 4542	99.49

**Table 2:** The mortality of hybrid groupers at different challenge concentrations (*n* = 3).

Group	*V. harveyi* GDH11385	Mixture of Isolated Pathogens	Number of Fish	7-Day Average Mortality
A	0 (1 × PBS)	1 × 10^5^ CFU/g	10	0%
B	1 × 10^3^ CFU/g	1 × 10^5^ CFU/g	10	3.33%
C	1 × 10^4^ CFU/g	1 × 10^5^ CFU/g	10	13.33%
D	1 × 10^5^ CFU/g	1 × 10^5^ CFU/g	10	46.67%
E	1 × 10^5^ CFU/g	0 (1 × PBS)	10	33.33%
CK	0 (1 × PBS)	0 (1 × PBS)	10	0%

## Data Availability

Not applicable.
